# Temperature Effects on the Structural Stability of EF_4_K Peptide Membranes: Insights into Mono- and Multilayer Architectures

**DOI:** 10.1021/acsmaterialsau.5c00214

**Published:** 2026-01-02

**Authors:** Karinna Mendanha, Douglas Xavier de Andrade, Guilherme Colherinhas

**Affiliations:** † Instituto de Física, Universidade Federal de Goiás, 74690-900 Goiânia, GO, Brazil; ‡ Instituto Federal de Educação Ciência e Tecnologia de Goiás, 74968-755 Aparecida de Goiânia, GO, Brazil

**Keywords:** molecular dynamics, peptide membrane, supramolecular assembly, temperature, hydrogen bond

## Abstract

Peptide nanostructures are versatile supramolecular systems with potential applications in biomaterials and nanotechnology, where stability emerges from the cooperative action of noncovalent interactions. In this study, we investigated the bola-amphiphilic peptide EF_4_K assembled into nanomembranes, focusing on the combined effects of temperature and multilayer organization. Molecular dynamics simulations were conducted at 250, 270, 300, 320, and 350 K in monolayer and multilayer configurations, allowing direct evaluation of peptide–peptide and peptide–solvent interactions. The results demonstrate that while the number of hydrogen bonds increases with temperature, their lifetimes decrease markedly, with reductions of nearly 79%. Peptide–solvent interactions weaken significantly, with losses of up to 90%, whereas multilayer assemblies partially compensate this destabilization by reinforcing peptide–peptide hydrogen bonds and van der Waals contacts. Electrostatic contributions between peptides remain stable and even strengthen in multilayers, indicating supramolecular reinforcement upon stacking. Confined water within multilayers exhibits longer hydrogen bond lifetimes despite a lower number of contacts, contrasting with the destabilization of hydration shells observed in monolayers at higher temperatures. These findings reveal that EF_4_K membranes undergo a redistribution of stabilizing forces under thermal stress, with multilayers achieving enhanced internal cohesion, thereby highlighting their potential as robust peptide-based nanomaterials.

## Graphical abstract

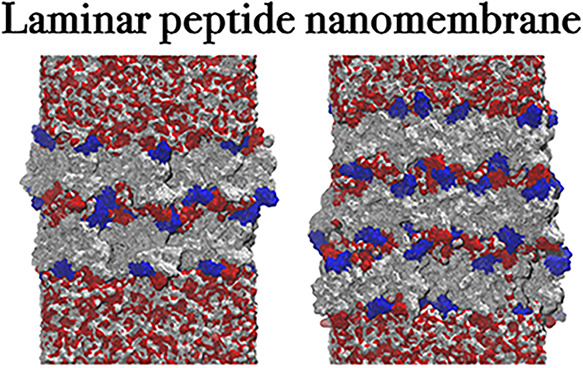

## 1 Introduction

Peptide nanostructures represent a class of supramolecular materials that emerge from the self-assembly of amino acid sequences. Previous studies have highlighted those noncovalent forces—including electrostatic, hydrophobic, hydrogen bonding, and aromatic stacking interactions—can drive the formation of β-sheets, nanotubes, nanofibrils, micelles, and membranes with a high degree of structural order.^1,2^ This structural diversity provides peptides with unique properties of biocompatibility, chemical stability, and the ability to modulate the final morphology of aggregates, features that are fundamental for biomedical and technological applications. The use of such structures has been widely reported in areas such as tissue regeneration, biosensors, antimicrobials, and molecular electronics. Nanomembranes and nanofibrils can act as scaffolds for cell growth, promote wound healing, encapsulate hydrophobic molecules, and even form selective channels for ion transport.^3–5^ Additionally, biodegradable ionic liquids have proven to be promising alternative media for maintaining the stability of these membranes, reinforcing perspectives for bioelectronic devices and sustainable supercapacitors.^6^ Thus, peptide nanostructures are consolidated as multifunctional platforms with potential integration into complex biomimetic systems.

From a molecular perspective, the choice of amino acid residues directly influences the stability and functionality of membranes. Polar residues favor the formation of polar zippers, increasing cohesion and flatness, while hydrophobic residues modulate flexibility and internal organization.^7,8^ The presence of specific residues such as histidine or phenylalanine adds distinctive capacities: metal coordination (Zn^2+^), antioxidant properties, and participation in π–π aromatic interactions.^4,9^ In systems such as A_6_R, spatial juxtaposition of peptides has been shown to be determinant for structural integrity and membrane permeability.^10^ Methodological advances in molecular modeling have enabled increasingly detailed analyses of the interactions responsible for self-assembly. Molecular dynamics simulations, both atomistic and coarse-grained, have revealed mechanisms of bilayer insertion, pore formation, supramolecular rigidity, and even the influence of ionic liquids on structural organization.^6,11,12^ Hybrid approaches combining classical molecular dynamics with quantum methods (DFT, GIAO-NMR, TD-DFT) further reinforce the importance of characterizing spectroscopic and electronic properties, broadening the understanding of the relationship between structure, function, and bioactivity.^9^ Another relevant line of research concerns the use of peptide nanostructures in biocatalysis and the inhibition of pathogenic biological processes. Fibrillar and micellar assemblies have been employed as platforms for positioning catalytic residues, functioning as biocatalysts in organic coupling reactions and green catalysis.^13^ In parallel, peptides designed to interact with viral proteins have demonstrated efficacy in inhibiting membrane fusion in viruses such as HIV-1 and SARS-CoV-2, evidencing the therapeutic potential of these nanostructures.^14^ Amphiphilic peptide micelles, in turn, have shown usefulness in targeted drug delivery and immunotherapy.^15^ The use of these complementary approaches supports a robust framework for the design of new peptide-based organic materials.

In this context, the present work investigates the stability and structural behavior of peptide membranes based on the EF_4_K peptide. Inspired by the structural logic proposed in earlier studies on β-sheet lamination^1^ and expanding the insights obtained in related investigations,^7,16^ this manuscript combines molecular dynamics simulations under different temperature conditions and arrangements (mono- and multilayer). The aim is to understand how hydrogen bonding, electrostatic forces, and van der Waals interactions contribute to the supramolecular robustness of these membranes, pointing to potential applications in biomaterials, nanotechnology, and bioinspired systems. In contrast to our previous studies on EF_4_K-based nanomembranes—which focused on single-temperature conditions or exclusively on monolayer architectures—the present work provides the first systematic temperature-dependent analysis of 1L, 2L, and 3L peptide membranes. By quantifying how electrostatic and van der Waals interactions, hydrogen-bond dynamics, and water confinement reorganize from 250 to 350 K, we reveal how supramolecular stability emerges from the interplay between thermal fluctuations and multilayer stacking. This comprehensive comparison across mono- and multilayer assemblies represents the key novelty of the present study.

## 2 Methodology

Initially, the EF_4_K peptide (E/Glu = Glutamic Acid; F/Phe = Phenylalanine; and K/Lys = Lysine) was constructed using the Packmol software^17^ and modeled with CHARMM36 force field.^18,19^ Subsequently, the “*gmx editconf*” tool from the Gromacs 2025 package^20,21^ was employed to organize the peptides into a membrane arrangement by stacking them sequentially. After building the initial structure, the system was solvated with water molecules modeled by the TIP3P force field^22,23^ and subjected to simulations under different temperature conditions and ambient pressure. To this end, five independent simulation boxes were prepared, each equilibrated at temperatures of 250, 270, 300, 320, and 350 K. It is important to note that temperatures above physiological conditions (e.g., 350 K) were included as part of a theoretical exploration of the thermal stability limits of EF_4_K membranes. Because the upper experimental tolerance of these assemblies has not yet been determined, simulations at elevated temperatures provide a controlled way to probe potential structural breakdown or resilience mechanisms. These predictions may help guide future experimental validation and identify the thermal robustness of the multilayer organization. The final configuration of the monolayer model (1L model) of the 300 K simulation was further used as a reference for the construction of more complex systems: the membrane obtained was stacked face-to-face, generating two new simulation boxes containing two layer (2L model) and three layer (3L model) structures. Figure 1 shows all systems generated for this work. The choice of the EF4K supramolecular arrangement follows the experimentally established membrane model proposed by Hu et al.,^1^ where electrostatically guided lamination and β-sheet untwisting lead to stable peptide nanosheets. This architecture has been validated extensively in experimental studies, and previous theoretical works employing the same EF_4_K sequence^16^ and the CHARMM36 force field^7–9,24–28^ have reproduced key experimental signatures, including hydration patterns, interfacial organization, and multilayer stability. The present all-atom representation therefore provides an appropriate balance between accuracy and computational efficiency, capturing the essential physical interactions responsible for membrane cohesion, hydration behavior, and stacking effects across temperatures. It is important to note that the multilayer arrangements examined here were not expected to emerge spontaneously during the simulations. Instead, their initial configurations follow the experimentally established laminated organization reported by Hu et al.,^1^ which proposes electrostatic-driven lamination and β-sheet untwisting as the mechanism underlying EF_4_K membrane stacking. The aim of the present simulations is therefore to evaluate the stability and thermodynamic behavior of this experimentally supported architecture, rather than to reproduce its self-assembly pathway

**Figure 1:**
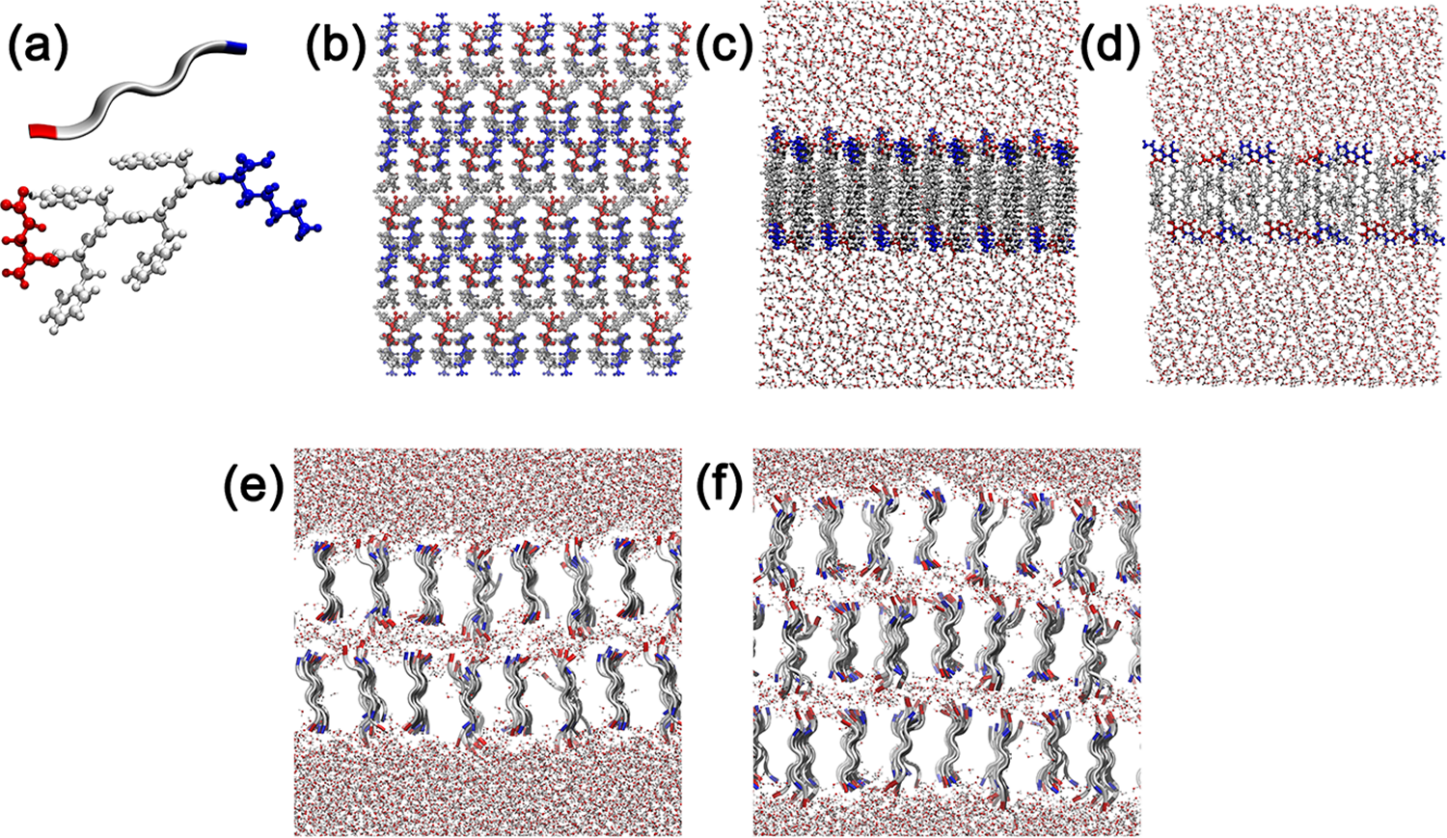
(a) EF_4_K peptide and ribbon representation colored by amino acid; (b) top view (*XY* plane); (c, d) side view (*Y* and *X* axes) of the initial peptide organization in the membrane (1L model); and Models with (e) two layers (2L model) and (f) three layers (3L model). Colors: E = red; F = white; K = blue; water molecules in red/white.

During classical molecular dynamics simulations, the temperature was maintained constant using the v-rescale thermostat^29^ with a coupling constant of 0.1 ps, and the pressure was stabilized by the semi-isotropic Parrinello–Rahman barostat^30^ with a coupling constant of 0.4 ps. For all MD simulations, Coulomb interactions were treated using the Particle Mesh Ewald (PME)^31^ method with a cutoff radius of 1.2 nm, while Lennard-Jones interactions were calculated with a cutoff of 1.2 nm. The simulations employed an integration time step of 0.001 ps to solve Newton’s equations of motion. We adopted a 1 fs time step to obtain smoother temporal resolution for dynamical analyses such as hydrogen-bond lifetimes, HB autocorrelation functions, and short-time scale fluctuations in membrane interactions. This refinement improves the numerical fidelity of fast dynamical observables without affecting the stability or equilibration behavior of the systems. To reach thermodynamic equilibrium, short sequential simulations alternating between the NVT and NPT ensembles were performed for approximately 50 ns. The NVT steps stabilize the system temperature and relax high-energy contacts without allowing fluctuations in the simulation box, while the NPT steps adjust the system density and box dimensions under the target pressure. This combined procedure ensures that both thermostat- and barostat-controlled variables converge properly. Once thermodynamic stability was verified, an additional 50 ns simulation in the NPT ensemble was carried out to fully relax the membrane structure under constant-pressure conditions before initiating the production phase. Only after this stage, and once equilibrium had been confirmed, the production simulations in the NPT ensemble were conducted for 100 ns. For statistical analyses of the studied properties, 50,000 configurations from the equilibrated trajectories were recorded. The LINCS^32^ algorithm was applied throughout all classical molecular dynamics’ simulations. Equilibration was monitored through the time evolution of the total potential energy and hydrogen-bond occupancy, representative convergence profiles for 1L, 2L, and 3L systems at 300 K are provided in the Supporting Information (Figures S1–S2). Table 1 shows the composition of the simulated structures, where single-layer membrane systems were kept identical across the simulations under different temperature conditions to facilitate comparison. Simulations were carried out using the Gromacs software (version 2023).^20,33^ Visualization and structural analyses were performed with the Visual Molecular Dynamics (VMD) program,^34^ and quantitative analyses (including potential energy, structural variations, and peptide dynamics) were obtained from the simulated trajectories.

**Table 1:** Composition of Membrane Structures Highlighting the Initial and Final Dimension (and Volume) of the Simulation Boxes and the Total Number of Atoms and Particles in Each Simulation

	composition				
system	#peptide	#water molecules	total atoms	initial box dimensions (nm)	initial and final volume (nm^3^)
1-Layer (1L)	72	9947	38,481	6.204; 6.882; 9.000	384.47; 378.41
2-Layer (2L)	144	11,345	51,315	6.825; 7.482; 12.482	636.86; 501.60
3-Layer (3L)	216	12,258	76,611	6.825; 7.482; 15.223	778.92; 618.98

The composition of the simulated system is presented in Table 1. To characterize the structural and dynamical behavior of EF_4_K peptide membranes, a series of analyses was conducted based on the molecular dynamics’ trajectories. The hydrogen bond dynamics between peptides and between peptides and water were evaluated using the geometric criterion established by Luzar and Chandler,^35–37^ considering a hydrogen bond present when the donor–acceptor distance was less than 3.5 Å and the donor–hydrogen–acceptor angle was below 30°. Nonbonded interaction energies, including Coulomb (electrostatic) and Lennard-Jones (van der Waals) contributions, were averaged over the equilibrated portion of the trajectory, reflecting the internal energetic stabilization of the system. In addition, conformational sampling of the peptide backbone was analyzed through Ramachandran plots,^38^ constructed from φ and ψ dihedral angles extracted from the trajectories.

## 3 Results and Discussion

### 3.1 Hydrogen Bonds, Lifetime, and Energy Analyses

The hydrogen bonds analyses shows that the temperature strongly modulated the hydrogen bonding network in EF_4_K 1L model, as shown in Table 2. Between peptides, the average number of hydrogen bonds increased slightly from 4.9 at 250 K to 5.4 at 350 K (∼10%), yet their lifetimes decreased from 1.55 to 0.33 ns (∼79%), and Δ*G* values dropped by ∼17%. This indicates that higher temperatures favor more frequent but less stable interactions, yielding a more dynamic and transient peptide–peptide network. In parallel, peptide–solvent interactions declined sharply at the same temperature interval: the number of hydrogen bonds decreased by ∼29%, lifetimes by ∼89%, and Δ*G* by ∼33%. Residue-level analyses confirmed that Glu and Lys residues lost ∼20–25% of their hydrogen bonds and nearly 90% of their lifetime, while Phe residue exhibited the largest relative reduction (∼62% fewer bonds, ∼84% shorter lifetimes). These data point to a progressive weakening of the interfacial hydration shell and destabilization of solvent-mediated contacts under thermal variations. In contrast, membrane stacking at 300 K reinforced supramolecular cohesion. The number of peptides–peptides hydrogen bonds increased by ∼13% in bilayers and ∼19% in 3L model relative to the 1L model, while lifetimes extended by ∼177% and ∼288%, respectively. At the same time, Δ*G* values increased by ∼13% and ∼16%. Although lower Δ*G* indicates weaker individual bonds, the larger number and longer persistence of peptide–peptide contacts in stacked systems highlight a collective stabilization effect: the integrity of the assembly is sustained not by the strength of single interactions but by the synergy of multiple, dynamically exchanging bonds.

**Table 2:** Average Number of Hydrogen Bonds, Lifetimes (τ), and Gibbs Free Energies (Δ*G*) for EF_4_K Systems at Different Temperatures and Stacking Configurations, Reported Per Peptide (*r* ≤ 0.35 nm and θ = 30°)^a^

		Pep-Pep			Pep-Sol			Glu-Sol			Phe-Sol			Lys-Sol		
	*T*	HB	*t*	Δ*G*	HB	*t*	Δ*G*	HB	*t*	Δ*G*	HB	*t*	Δ*G*	HB	*t*	Δ*G*
1L	250	4.9	1.55	22.7	14.2	0.18	17.4	7.8	0.09	15.8	2.1	1.95	23.3	4.3	0.11	16.3
	270	4.9	1.01	21.7	13.5	0.11	16.2	7.6	0.06	14.6	1.7	1.37	22.4	4.2	0.07	15.0
	300	5.3	0.69	20.7	11.9	0.05	14.1	6.8	0.03	12.6	1.2	0.81	21.1	3.9	0.03	13.3
	320	5.2	0.57	20.3	11.4	0.03	12.9	6.6	0.02	11.5	1.0	0.55	20.2	3.8	0.02	12.3
	350	5.4	0.33	18.9	10.1	0.02	11.6	5.9	0.01	10.4	0.8	0.32	18.8	3.4	0.01	11.2
2L	300	6.0	1.91	23.3	9.5	0.30	18.7	5.5	0.21	17.8	0.9	2.02	23.4	3.1	0.20	17.7
3L	300	6.3	2.68	24.1	8.3	0.56	20.2	4.7	0.43	19.5	0.9	2.12	23.5	2.7	0.28	18.5

a Lifetimes (τ) are given in nanoseconds (ns) and Δ*G* values in kJ·mol^–1^.

This opposite trend—a higher number of hydrogen bonds accompanied by shorter lifetimes—reflects an entropically driven regime at elevated temperatures. As the thermal energy increases, the peptide backbone explores a broader ensemble of configurations, leading to more frequent transient approaches between donor and acceptor groups. This enhances the instantaneous probability of H-bond formation, thereby increasing the average count. However, the same increase in backbone fluctuations and side-chain mobility promotes rapid disruption and reformation events, reducing the stability of individual hydrogen bonds. Thus, the system shifts toward fast HBs exchange kinetics, where dynamic rearrangement is entropically favored over long-lived interactions.

Peptide–solvent interactions followed the opposite pattern. Hydrogen bond numbers decreased by ∼20% in bilayers and ∼30% in 3L model relative to the 1L model, but the remaining contacts became substantially more persistent, with lifetimes increasing by more than 6-fold and Δ*G* values increasing by up to ∼43%. This redistribution suggests that interfacial water molecules, though fewer in number, are selectively retained in confined microenvironments, forming more long-time interactions, but energetically weaker. Residue-specific behavior corroborates these observations: Glu and Lys residue retained fewer water molecules but exhibited increases in hydrogen bond lifetime, while Phe residue showed reduced hydration consistent with aromatic packing and water exclusion in multilayered assemblies. Altogether, these findings demonstrate that the stability of EF_4_K membranes under thermal variations arises not from maintaining hydration in monolayers but from their intrinsic ability to self-organize into stacked supramolecular structures, where cooperative peptide–peptide interactions and confined water contacts ensure robust cohesion. Figure 2 shows the hydrogen bond, hydrogen bond lifetime and Gibbs’ energy for all systems.

**Figure 2:**
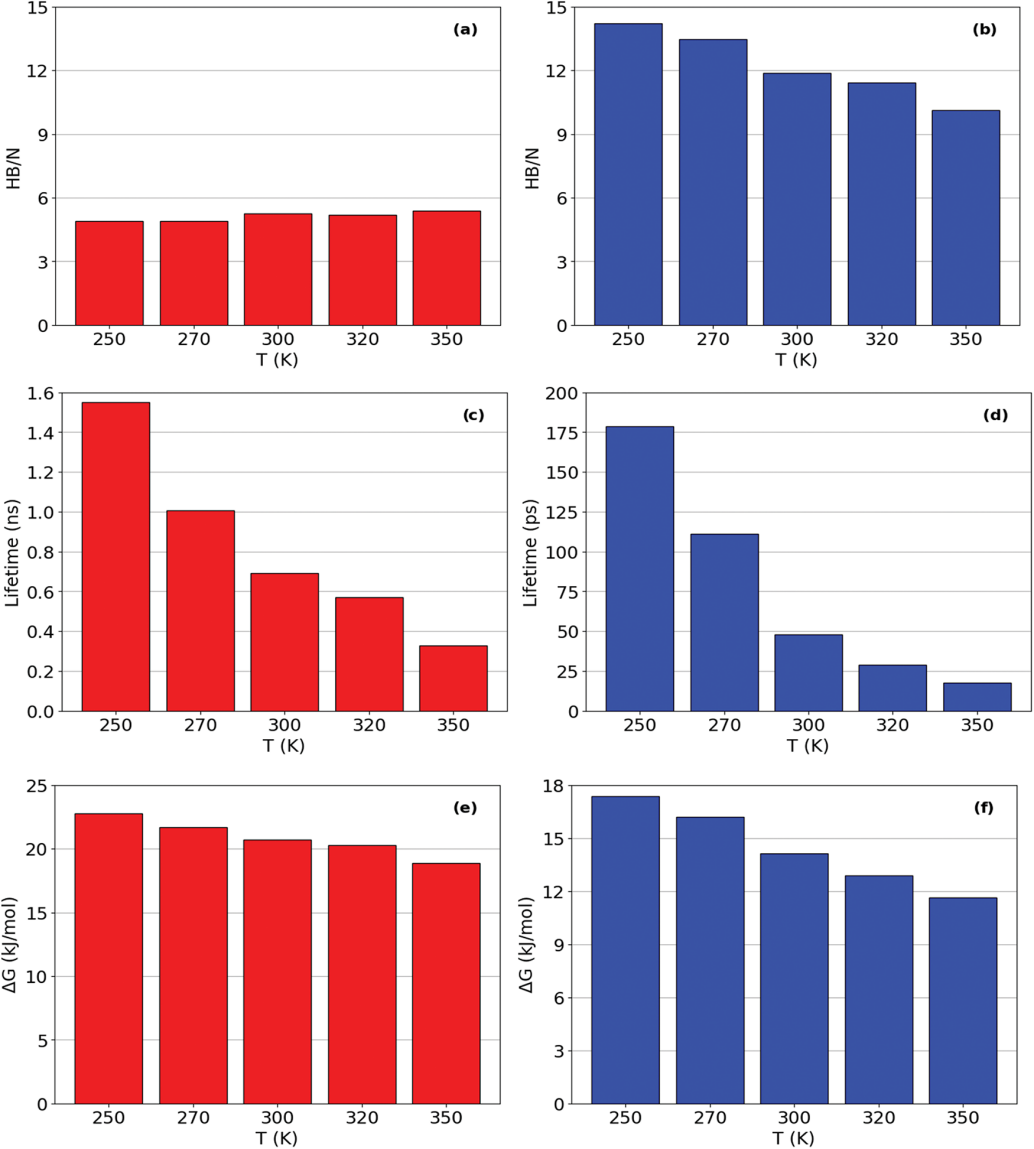
Average number of hydrogen bond, hydrogen bond’s lifetime and Gibbs Energy for hydrogen bond rupture for 1L model in all temperature simulated. (a, c, and e) peptide–peptide interaction; and (b, d, and f) peptide-water interaction.

To assess the sensitivity of the hydrogen-bond (HB) analysis to the adopted geometric criteria, all statistics were recalculated using a less restrictive donor–H–acceptor angular cutoff of 40°, in comparison with the standard 30° definition. As expected, relaxing the angular criterion leads to a systematic increase in the absolute number of detected hydrogen bonds and a modest increase in their associated lifetimes and Δ*G* values across all systems, reflecting the inclusion of more distorted yet still physically relevant HB geometries. Despite these quantitative shifts, all qualitative trends remain fully preserved. In monolayer membranes, peptide–peptide hydrogen bonds continue to exhibit only a weak temperature dependence in number, while their lifetimes decrease markedly with increasing temperature, whereas peptide–water hydrogen bonds show a clear reduction in both population and stability upon heating. Crucially, the stabilizing effect of stacking is maintained under the 40° criterion: both 2L and 3L systems display higher numbers of peptide–peptide hydrogen bonds, significantly longer lifetimes, and slightly more favorable Δ*G* values compared to the monolayer at the same temperature, while peptide–water interactions are reduced in number but become more persistent. The relative enhancement observed upon stacking is even marginally amplified with the 40° cutoff, as multilayer assemblies’ benefit from a larger fraction of interlayer hydrogen bonds with nonideal angular geometries. These results demonstrate that the conclusions regarding hydrogen-bond reorganization, thermal destabilization of hydration shells, and stacking-induced supramolecular stabilization are robust with respect to the specific geometric definition of hydrogen bonding. The complete data set obtained using the 40° angular cutoff is provided in the Supporting Information (Table S1).

### 3.2 Coulomb and Lennard-Jones Interaction Energy

The analysis of Coulomb interactions revealed two distinct behaviors: while intrinsic peptide–peptide contributions remained highly stable across the studied temperature range, peptide–solvent interactions were progressively weakened, as represented in Table 3. In 1L model, the peptide–peptide term exhibited strongly negative values, from −2406 kJ/mol per peptide at 250 K to −2442 kJ/mol at 350 K, with only ∼1.5% variation. This result indicates that electrostatic cohesion between peptide chains remains robust even under thermal variation, ensuring structural stability within the membrane. In contrast, peptide–solvent electrostatic contributions were markedly reduced by heating. The mean peptide–water value decreased from −644 kJ/mol at 250 K to −469 kJ/mol at 350 K (∼27% reduction), reflecting weaker coupling to water at higher temperatures. Residue-specific contributions followed the same trend: Glu decreased by ∼24% (−380 to −290 kJ/mol), Lys by ∼22% (−196 to −153 kJ/mol), and Phe by ∼60% (−67.6 to −26.8 kJ/mol). Unlike hydrogen bonds, which are directional and require favorable geometry, Coulomb interactions energy depend only on distance. Their reduction therefore does not reflect a loss of angular alignment but rather diminished residence of water molecules near polar and aromatic groups, leading to a lower overall electrostatic contribution from the solvent.

**Table 3:** Average Coulomb Interaction Energies of EF_4_K Systems at Different Temperatures and Stacking Configurations, Reported Per Peptide^a^

system	*T* (K)	peptide–peptide	peptide–water	Glu-water	Phe-water	Lys-water
1L	250	–2406 ± 6	–644 ± 11	–380 ± 7	–68 ± 2	–196 ± 6
	270	–2409 ± 9	–619 ± 16	–371 ± 8	–56 ± 3	–192 ± 7
	300	–2428 ± 9	–541 ± 17	–331 ± 9	–40 ± 3	–171 ± 8
	320	–2430 ± 9	–535 ± 15	–328 ± 9	–34 ± 3	–172 ± 7
	350	–2442 ± 10	–469 ± 19	–290 ± 10	–27 ± 3	–153 ± 8
2L	300	–2491 ± 5	–411 ± 8	–166 ± 5	–17 ± 1	–84 ± 4
3L	300	–2516 ± 3	–352 ± 5	–111 ± 3	–11 ± 1	–56 ± 3

a Values are given in kJ·mol^–1^, per peptide.

Stacking further reinforced this redistribution. At 300 K, the peptide–peptide contribution became more negative, strengthening from −2430 kJ/mol in 1L model to −2491 kJ/mol in 2L model (∼2.5%) and −2516 kJ/mol in 3L model (∼3.5%). Conversely, the peptide–water contribution decreased sharply, from −535 kJ/mol in the 1L model (320 K) to −411 kJ/mol in the 2L model (−23%) and −352 kJ/mol in the 3L model (−34%). Residue-level analyses revealed even larger decreases: Glu lost ∼50% of its electrostatic stabilization in 2L model and ∼66% in 3L model; Lys decreased by ∼51% and ∼67%, respectively; and Phe by ∼57% and ∼72%. These results suggest that stacking shifts the balance of electrostatic stabilization from the solvent to the peptide framework itself. This behavior is consistent with the formation of a hydrophobic supramolecular core, in which aromatic and even charged residues become partially shielded from water. In this configuration, stabilization is no longer predominantly solvent-driven but is reinforced by intramembrane salt bridges and cooperative contacts. Importantly, while hydrogen bonds rely on strict angular constraints and directional alignment, Coulomb interactions benefit simply from dense packing, since proximity alone is sufficient to sustain attraction. Overall, the analysis of Coulomb interactions energy demonstrates that the stability of EF_4_K membranes arises from a reorganization of the balance between solvent contributions and peptide–peptide forces. Increasing temperature weakens water-mediated stabilization without disrupting the intrinsic electrostatic network, whereas stacking consolidates internal stabilization and favors the emergence of a solvent-shielded core maintained by nondirectional electrostatics. This structural reorganization confers enhanced supramolecular robustness and resilience to thermal fluctuations.

Lennard-Jones interactions energy between peptides were strongly stabilizing across all systems, with values ranging from −239 kJ/mol at 250 K to −245 kJ/mol at 350 K in 1L model (∼2.5% more negative), as presented in Table 4 (all average values per peptide). This relative insensitivity to temperature suggests that dispersion forces between peptide chains remain robust even under thermal stress, providing a persistent contribution to membrane cohesion. By contrast, peptide–solvent contributions weakened progressively with increasing temperature. The peptide–water term decreased from −31 kJ/mol at 250 K to −12 kJ/mol at 350 K (∼60% reduction), indicating that water molecules become less engaged in dispersive stabilization as the hydration network is disrupted. Among individual residues, Phe displayed the strongest stabilizing effect in monolayers (−40 kJ/mol at 250 K), but this value was reduced to −26 kJ/mol at 350 K (∼35%), consistent with the progressive exclusion of water from aromatic regions. Glu, in contrast, exhibited consistently positive values (+12 to +13 kJ/mol), while Lys fluctuated near zero and became slightly positive at higher temperatures. This behavior can be understood in terms of the Lennard-Jones potential energy: because Glu and Lys are strongly stabilized by electrostatic interactions, their charged groups are driven into closer contact with water molecules, reaching distances shorter than the optimal for dispersion. In this repulsive regime of the potential, the vdW contribution becomes positive, reflecting an energetic penalty that is nonetheless compensated by favorable Coulomb stabilization. Stacking at 300 K further reinforced this trend by strengthening intramembrane stabilization and reducing solvent contributions. The peptide–peptide interaction became more negative, from −246 kJ/mol in 1L model (320 K) to −253 kJ/mol in 2L model (+2.5%) and −255 kJ/mol in 3L model (+3.5%). In parallel, the peptide-water contribution decreased from −13 kJ/mol in 1L model to −10 kJ/mol in 2L model (−21%) and −7 kJ/mol in 3L mode (reduction of the ∼44%). Phe lost ∼70% of its stabilizing contribution (−28 to −9 kJ/mol), consistent with water exclusion upon dense packing of aromatic side chains. Glu and Lys, meanwhile, shifted toward values closer to zero in multilayered systems, reflecting reduced solvent exposure and partial sequestration of charged groups within the peptide core. Overall, LJ interactions act as a structural pillar of EF_4_K assemblies, largely insensitive to temperature and enhanced by stacking. At the same time, water contributions diminish as the interface is progressively dehydrated, particularly around aromatic residues. For charged residues such as Glu and Lys, positive vdW values indicate repulsion arising from close-range overlap, driven by strong electrostatics that force groups into unfavorable regions of the Lennard-Jones potential. These observations underscore the complementary role of vdW forces within the balance of hydrogen bonds and electrostatics, supporting the emergence of a hydrophobic supramolecular core that ensures cohesion of stacked membranes and resilience against thermal fluctuations.

**Table 4:** Average van der Waals Interaction Energies of EF_4_K Systems at Different Temperatures and Stacking Configurations, Reported Per Peptide^a^

system	*T* (K)	peptide–peptide	peptide–water	Glu-water	Phe-water	Lys-water
1L	250	–239 ± 2	–31 ± 3	12 ± 2	–40 ± 1	–3 ± 2
	270	–241 ± 2	–23 ± 3	13 ± 2	–34 ± 1	–1 ± 2
	300	–245 ± 9	–18 ± 2	11 ± 2	–28 ± 1	–1 ± 2
	320	–246 ± 2	–13 ± 3	11 ± 2	–26 ± 1	0.4 ± 1.5
	350	–245 ± 3	–12 ± 3	11 ± 2	–26 ± 1	0.4 ± 1.5
2L	300	–253 ± 1	–10 ± 2	6 ± 1	–13 ± 1	0.2 ± 0.9
3L	300	–255 ± 1	–7 ± 1	4 ± 1	–9 ± 0.4	0.1 ± 0.5

a Values are given in kJ·mol^–1^, per peptide.

Taken together, the quantitative trends from hydrogen-bond statistics and interaction energies demonstrate that multilayer stabilization is predominantly thermodynamic in origin. At 300 K, stacking increases the number of peptide–peptide HBs from 5.3 in the 1L membrane to 6.0 and 6.3 in the 2L and 3L models (∼13% and ∼19% gains), while the corresponding lifetimes rise from 0.69 to 1.91 ns and 2.68 ns, i.e., almost three- to 4-fold enhancements. In parallel, the Gibbs free energy associated with hydrogen-bond rupture increases from 20.7 kJ·mol^–1^ in 1L to 23.3 kJ·mol^–1^ and 24.1 kJ·mol^–1^ in 2L and 3L, indicating more persistent and cooperative interlayer contacts. These hydrogen-bond trends are fully consistent with the interaction-energy analysis: the peptide–peptide Coulomb contribution becomes more favorable upon stacking (from −2428 ± 9 to −2491 ± 5 and −2516 ± 3 kJ·mol^–1^ per peptide), whereas the peptide–water term is strongly attenuated (from −541 ± 17 to −411 ± 8 and −352 ± 5 kJ·mol^–1^), reflecting partial dehydration of the interlamellar region. This redistribution of stabilization from solvent-mediated to intramembrane interactions points to an enthalpic gain associated with tighter peptide packing, complemented by an entropic contribution arising from the release of water molecules from the hydrophobic core. Thus, multilayer stability emerges from a cooperative thermodynamic mechanism combining stronger interpeptide interactions with dehydration-assisted entropy, rather than from kinetic confinement alone.

To refine the energetic interpretation of stacking stabilization, the peptide–peptide interaction energy was decomposed into intralayer and interlayer Coulomb and Lennard-Jones contributions for the 1L, 2L, and 3L assemblies. The layer-resolved analysis reveals that intralayer interactions remain strongly stabilizing and nearly invariant upon stacking, with Coulomb energies close to −2445 kJ·mol^–1^ per peptide and Lennard-Jones contributions around −248 to −251 kJ·mol^–1^ per peptide, indicating that the intrinsic cohesion within each peptide sheet is preserved in multilayer systems. In contrast, stabilization arising specifically from stacking originates from interlayer interactions. In the 2L system, the interaction between adjacent layers (1–2) contributes −46.45 kJ·mol^–1^ per peptide from Coulomb interactions and −3.11 kJ·mol^–1^ from Lennard-Jones terms, evidencing a non-negligible energetic gain associated with electrostatically driven lamination. A similar trend is observed in the 3L system, where nearest-neighbor interlayer contacts (1–2 and 2–3) contribute −46.34 and −44.51 kJ·mol^–1^ per peptide in Coulomb energy, respectively, complemented by Lennard-Jones contributions of −4.25 and −5.28 kJ·mol^–1^. As expected from the spatial separation, no direct energetic coupling is detected between nonadjacent layers (1–3). These results demonstrate that multilayer stabilization is dominated by short-range, nearest-neighbor electrostatic interactions, with van der Waals forces providing a secondary but consistent contribution. Importantly, the cumulative effect of these interlayer interactions leads to a progressive reinforcement of supramolecular cohesion as the number of stacked layers increases, confirming that stacking enhances membrane stability primarily through cooperative Coulomb-driven contacts across adjacent peptide slabs rather than through changes in intralayer energetics.

To complement the dynamical analyses, mean square displacement (MSD) calculations were performed for the peptide backbone and side chains in the 1L, 2L, and 3L systems at 300 K, allowing the extraction of effective diffusion coefficients for each assembly. The results reveal a clear reduction in peptide mobility upon stacking. In the monolayer system, peptides exhibit a diffusion coefficient of *D* = 1.78 × 10^–8^ cm^2^·s^–1^, reflecting higher translational freedom at the solvent-exposed interface. Upon stacking into a bilayer, peptide mobility decreases by nearly 1 order of magnitude, with diffusion coefficients of *D* = 1.18 and 1.22 × 10^–6^ cm^2^·s^–1^ for layers 1 and 2, respectively, indicating that interlayer contacts effectively constrain lateral and out-of-plane fluctuations. This restriction becomes even more pronounced in the three-layer system, where diffusion coefficients further decrease to *D* = 5.77, 4.68, and 5.81 × 10^–7^ cm^2^·s^–1^ for layers 1, 2, and 3, respectively. The comparable mobilities of the outer layers and the slightly reduced diffusion of the central layer reflect the stronger confinement experienced by peptides embedded within the multilayer stack. Overall, the progressive suppression of peptide diffusion with increasing layer number is fully consistent with the longer HBs lifetimes, more favorable peptide–peptide interaction energies, and enhanced structural compaction observed in multilayer assemblies.

### 3.3 Mass Density Profile

The mass density profiles along the *z*-axis provide structural support for the interaction trends observed across temperatures and stacking configurations. In monolayers (1L model), Figure 3, the peptide core remains centered and well-defined throughout the temperature range, yet the peaks become broader and slightly reduced in height at higher temperatures. This reflects in a slightly increase in structural flexibility and enhanced interfacial fluctuations. These changes are consistent with the progressive weakening of peptide–water interactions as observed by the reduction in the number of hydrogen bonds, Coulomb, and LJ energy interactions. In contrast, peptide–peptide interactions remain largely unaffected forming a well-stable core at all temperatures evaluated. Stacking produces a markedly different organization. In 2L and 3L models, Figure 4, sharper and more compact peptide density peaks emerge, separated by confined interlamellar water compartments. This arrangement coincides with enhanced peptide–peptide interactions and a global reduction in solvent contributions. However, the confined water exhibits higher hydrogen bond lifetimes and more structured behavior, even under reduced hydration. This behavior is consistent with the restricted mobility of the interlamellar water population. Because the region between peptide layers is partially hydrophobic and sterically confined, water exchange with the bulk becomes limited, leading to longer residence times and consequently longer hydrogen-bond lifetimes. Even though fewer water molecules are present in the interlayer space, those that remain tend to be trapped for extended periods relative to surface-exposed water in the 1L model. This reduced exchange dynamics explains why interlamellar water forms longer-lived hydrogen bonds despite the decrease in overall hydration. Overall, the density profiles demonstrate that heating in monolayers increases interfacial flexibility and reduces hydration, thereby weakening solvent-mediated stabilization. By contrast, stacking favors the formation of a multilayered hydrophobic core, where stability arises primarily from tighter peptide packing and the selective retention of water molecules in confined microenvironments. This structural reorganization shifts the balance of stabilization from water-exposed contacts to intrinsic interactions within the peptide network, conferring greater supramolecular robustness and resilience to thermal fluctuations. Together, these mass density profiles offer a direct structural visualization of how water reorganizes along the membrane interface. The gradual broadening or sharpening of peptide and water density regions across temperatures and stacking configurations makes the evolution of the hydration shell explicit, providing a clear picture of how interfacial water becomes redistributed, confined, or released depending on the supramolecular organization. Additionally, to further resolve the structural contribution of each slab within the multilayer assemblies, we computed the mass-density profile of each peptide layer independently in the 2L and 3L models. This layer-resolved analysis, Figure 4c,4d, demonstrates that each slab maintains a well-defined and compact density peak, with minimal overlap between adjacent layers. The profiles highlight how stacking produces a more sharply organized architecture, where each peptide sheet contributes to the overall supramolecular ordering while preserving its intrinsic packing density.

**Figure 3:**
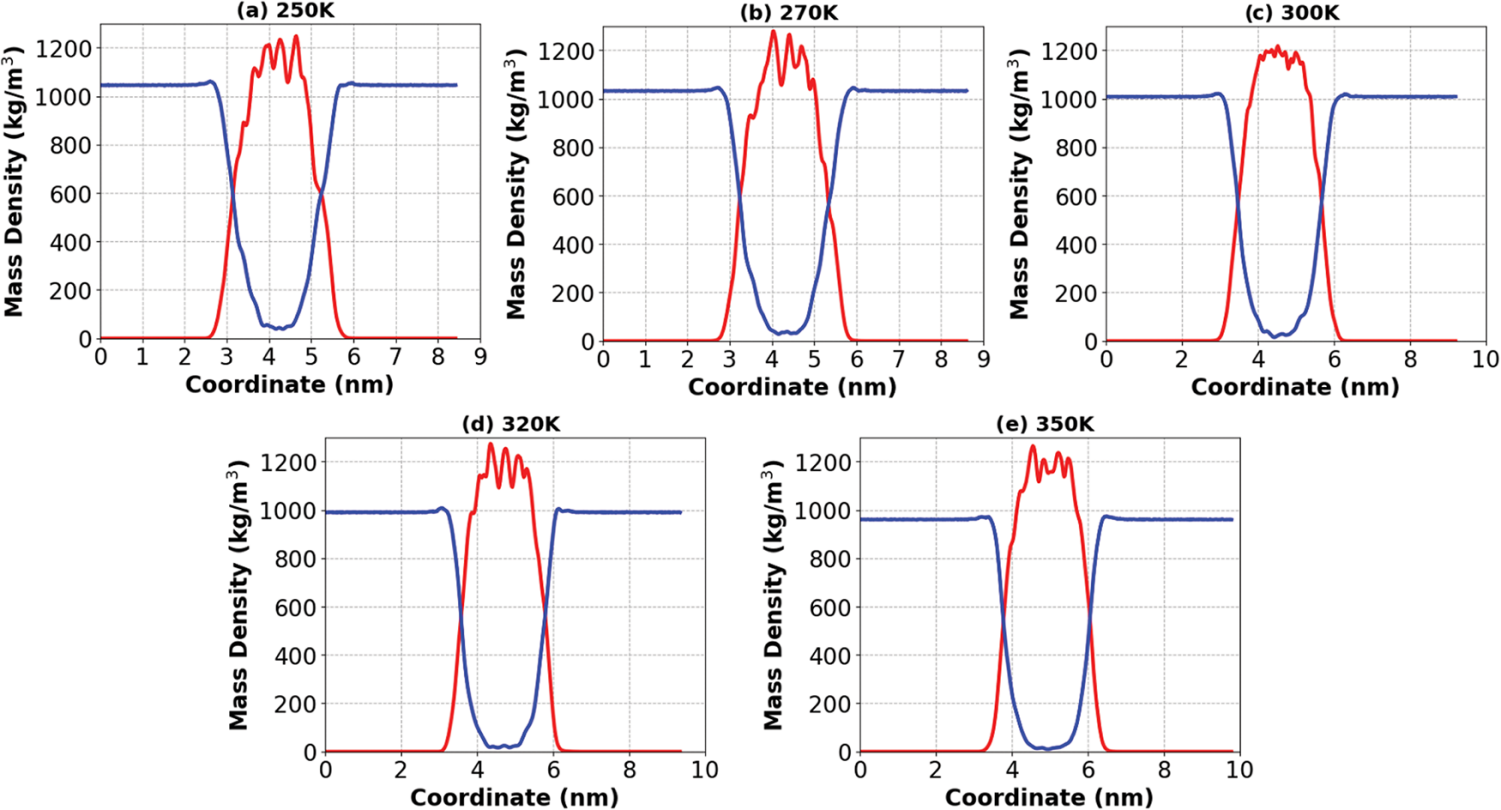
Mass density profiles of EF_4_K monolayers at different temperatures along the *z*-axis. Red curves represent peptide density profile and blue curves represent water density profile. (a) 250 K; (b) 270 K; (c) 300 K; (d) 320 K; and (e) 350 K.

**Figure 4:**
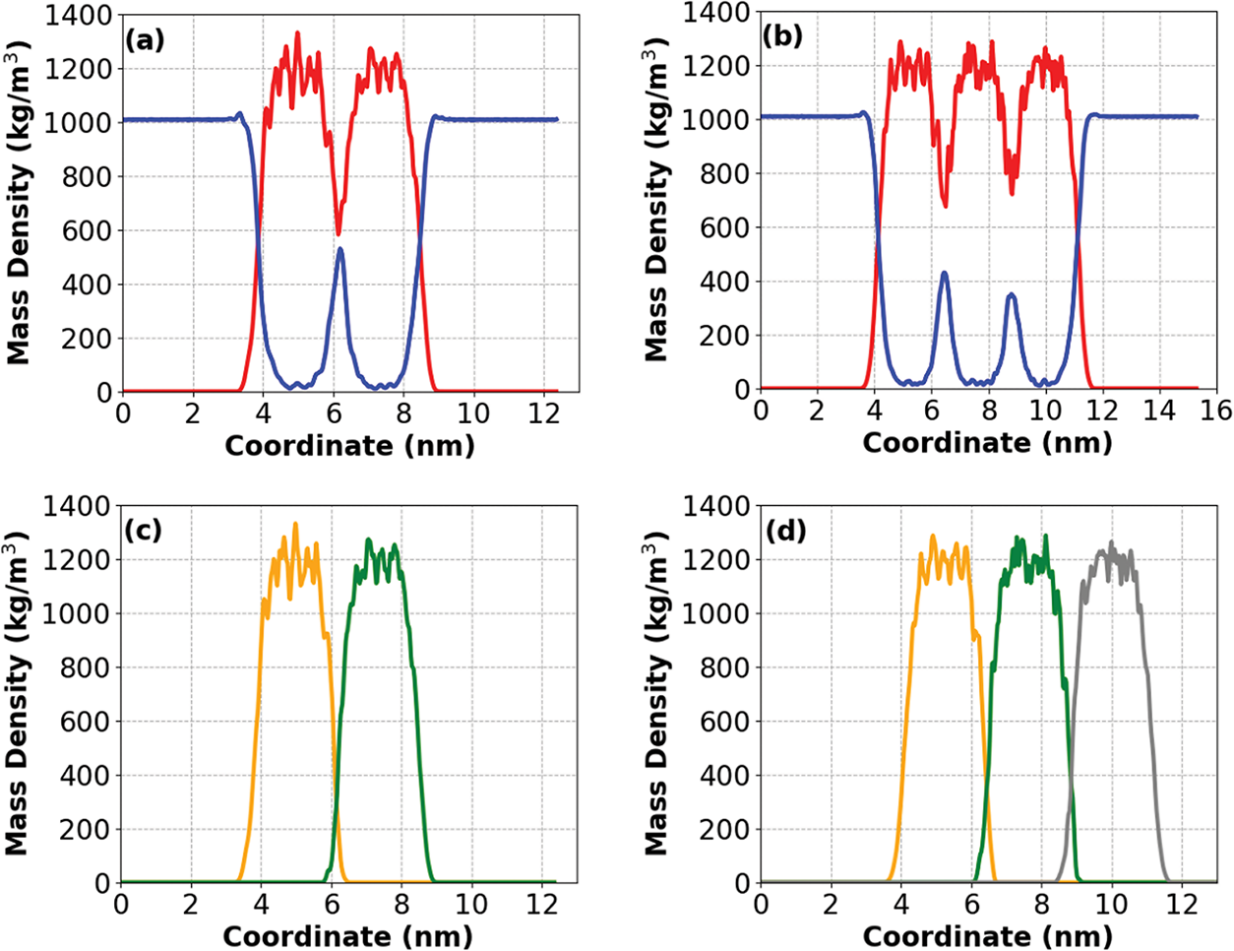
Mass density profiles (for *z* box length) of EF_4_K (a) 2L and (b) 3L models at 300 K. Red curves represent peptide density profile and blue curves represent water density profile. (c, d) Demonstrate the same representation as (a, b) but highlighting the mass density profile of each membrane layer individually, making it possible to assess the contact between each peptide layer.

In addition to the qualitative features of the density profiles, the distance between the intersections of the water and peptide mass density curves provides an estimate of the effective membrane thickness. In the 1L model, this thickness shows a slight expansion with increasing temperature (3.81 nm for 250 K and 3.88 nm for 350 K), consistent with the broader peptide peaks and enhanced interfacial fluctuations observed at higher thermal conditions. For the multilayered systems (2L and 3L), the measured thickness of each peptide slab at 300 K closely matches the value obtained for the 1L system at the same temperature, although the overall effective thickness of the stack is naturally increased by the additional layers. This indicates that the fundamental packing density of each layer remains preserved, while the multilayer arrangement introduces confined interlamellar water regions that further contribute to stability. To complement this structural descriptor, the peptide surface density can be calculated as the number of peptides per nm^2^, obtained by dividing the total number of peptides in the simulation box by the area of the membrane in the *XY* plane. This analysis reveals a weak dependence on temperature for the 1L system (1.66 peptide/nm^2^ at 250 K and 1.78 peptide/nm^2^ at 350 K), as slight thermal expansion of the box dimensions at higher temperatures reduces the peptide density per unit area. In multilayer systems, however, the peptide density per nm^2^ remains comparable (1.78 peptide/nm^2^) to that of the 1L system at 300 K (1.75 peptides/nm^2^), reinforcing that stacking preserves the intrinsic lateral organization of the peptides while simultaneously enhancing vertical supramolecular cohesion.

Figure 5 shows the mass density profile along the *y*-axis for the 1L, 2L, and 3L models, while Figure 6 highlights the final configurations of the structures in the *x*- and *y*-axis directions. As observed in both figures, there is a strong alignment of the peptide lamellae along the *x*-axis, with spacing occurring along the *y*-axis. This alignment is maintained with increasing temperature in the 1L models as well as in the stacked membranes. The average distance between each peptide lamella is 0.480–0.634 nm in the 1L model (depending on the temperature at which the structure was obtained) and 0.557 and 0.505 nm in the 2L and 3L models, respectively. These results demonstrate the high degree of ordering and structural compaction of these nanomembranes. For comparison, experimental results describe lamellar spacing of peptide β-sheets close to 0.86 nm,^26^ which is consistent with our theoretical findings. The structural descriptors derived from the mass density profiles and the multilayered organization of EF_4_K membranes provide insights that go beyond fundamental characterization and suggest potential technological applications. First, the confirmation of stable multilayered structures from peptide and water density distributions, combined with the energetic and thermal robustness demonstrated by the systems, highlights their suitability as biomaterial scaffolds for tissue engineering, where long-term stability and controlled mechanical flexibility may be required. Importantly, these computational results are consistent with experimental reports describing peptide membranes as intrinsically stable when forming stacked lamellar arrangements, further reinforcing the relevance of the observed supramolecular organization. Second, the observation of confined interlamellar water indicates that EF_4_K multilayers can act as selective membranes, retaining water in structured microenvironments alternating with regions that limit hydration (the peptide bulk of the layers), a feature that may also be exploited in separation processes or biomimetic filtration devices. Finally, the highly compact peptide packing and the structural robustness of multilayered systems provide an intrinsic barrier to permeability, suggesting potential for drug encapsulation and/or controlled release. Taken together, these findings position EF_4_K nanomembranes not only as supramolecular assemblies stable under thermal stress but also as a versatile biological platform for applications in biomaterials and/or nanomedicine.

**Figure 5:**
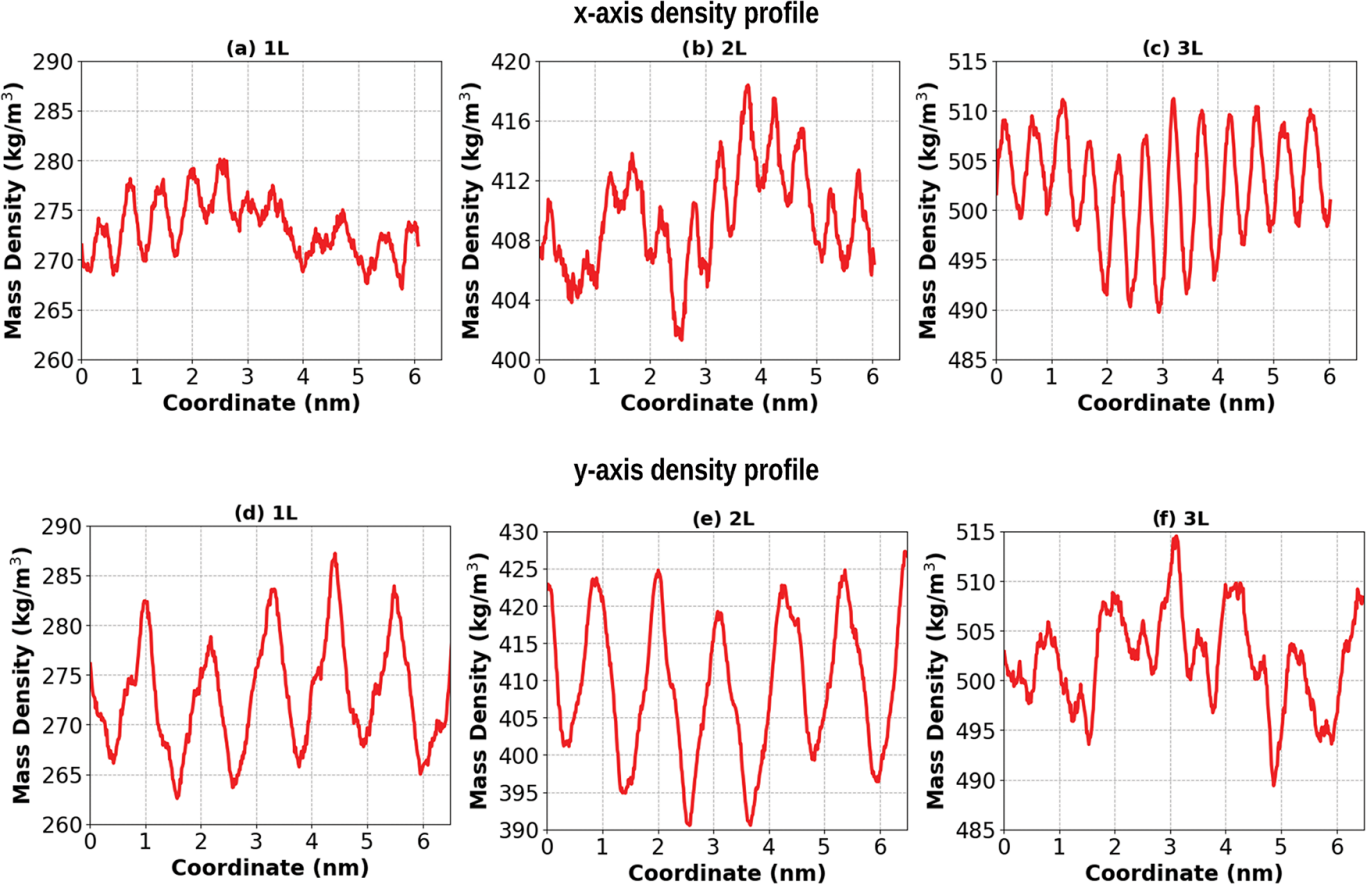
Mass density profiles (for *x* and *y* box length) of EF_4_K (a, d) 1L, (b, e) 2L, and (c, f) 3L models at 300 K. Red curves represent peptide density profile.

**Figure 6:**
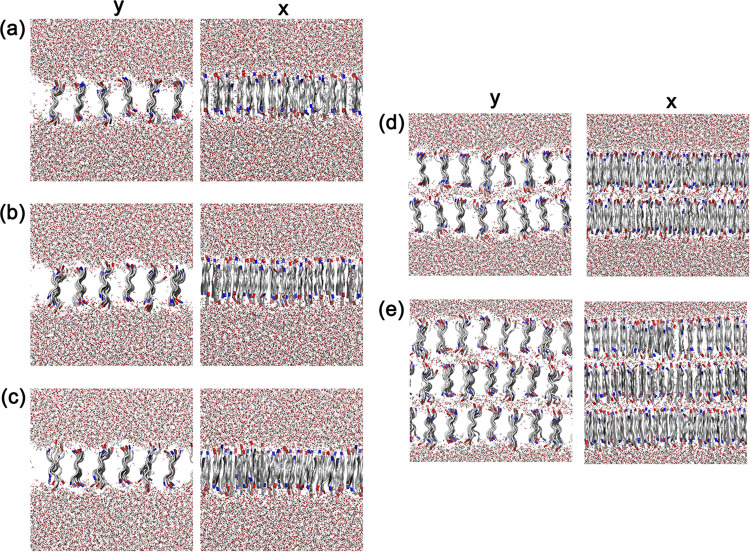
Lateral demonstration (*y* and *x* axes) of the final configuration of the nanostructures simulated. In yellow we shows the hydrogen bonds between peptide–peptide. (a) EF_4_K 1L model at 250 K; (b) EF_4_K 1L model at 300 K; (c) EF_4_K 1L model at 350 K; (d) EF_4_K 2L model at 300 K; and (e) 3L model at 300 K.

### 3.4 Ramachandran Plots

The Ramachandran plots presented in Figure 7 reveal a dominant conformational preference for the EF_4_K peptide toward the β-sheet region, characterized by angular clustering near φ ≈ −120° and ψ ≈ +120°. In the single-layer membrane at 300 K, the residues exhibit a highly concentrated distribution within this canonical β-sheet region, demonstrating the intrinsic propensity of EF_4_K to stabilize extended chain conformations and form planar supramolecular arrangements. The compactness of the distribution indicates a well-ordered backbone geometry, consistent with the lamellar organization observed in the structural analyses and hydrogen-bonding profiles.

**Figure 7:**
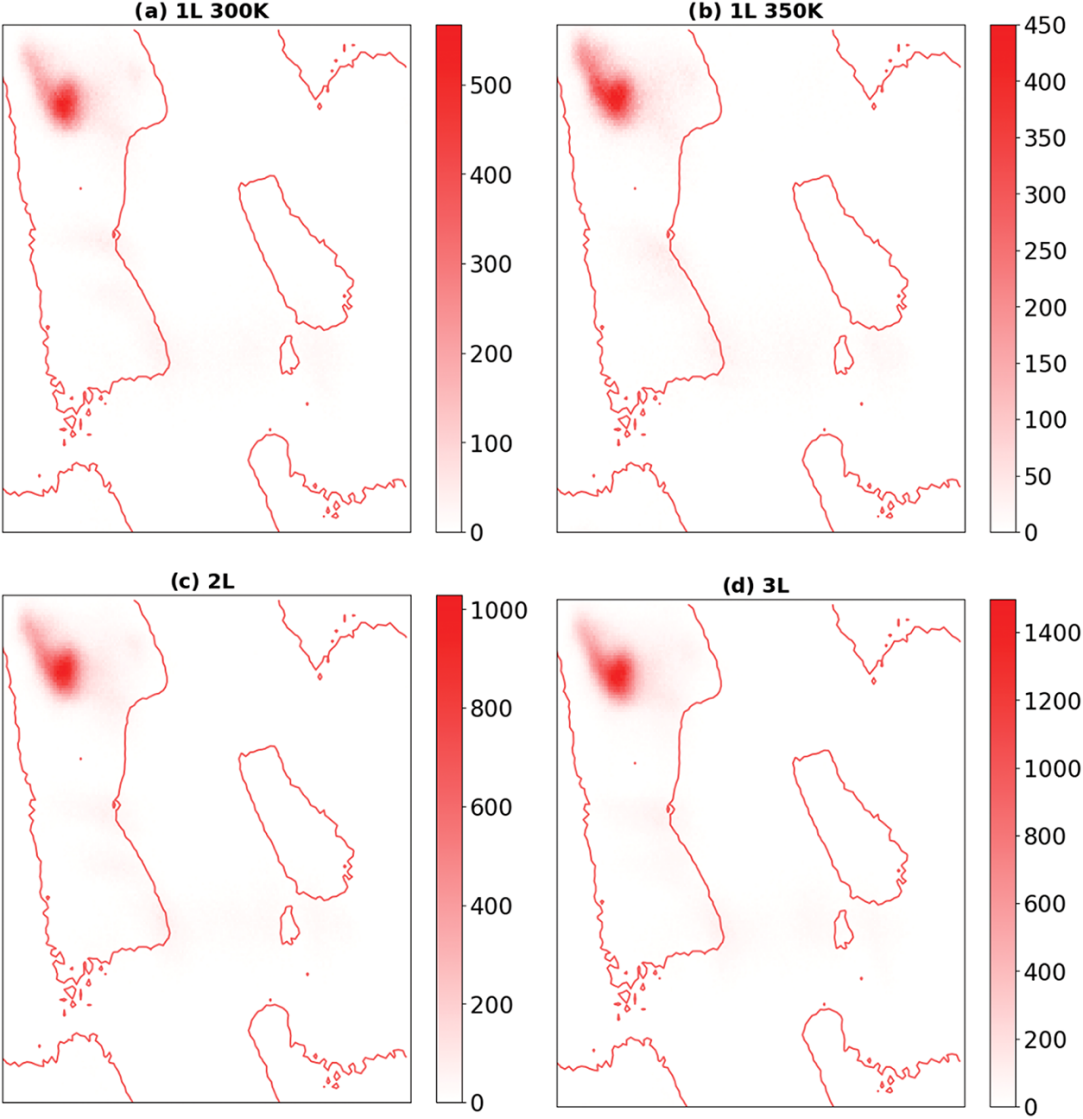
Ramachandran plots of EF_4_K peptide backbone dihedral angles (φ, ψ) under different thermal and organizational conditions. (a) Single-layer (1L) membrane at 300 K, demonstrating strong clustering in the β-sheet region, indicative of ordered lamellar conformation. (b) Single-layer (1L) membrane at 350 K, showing a modest increase in conformational dispersion while preserving the dominant β-sheet signature. (c) Two-layer (2L) membrane at 300 K, where enhanced confinement and interpeptide interactions lead to sharper localization of dihedral angles within the β-sheet basin. (d) Three-layer (3L) membrane at 300 K, exhibiting the highest degree of conformational restriction and β-sheet stabilization among the systems analyzed. Collectively, the plots confirm that EF_4_K peptide membranes maintain extended β-sheet–like conformations across thermal fluctuations and increasingly compact supramolecular architectures, consistent with the enhanced structural ordering observed in stacked systems.

Upon increasing the temperature to 350 K in the monolayer system, a modest broadening of the conformational distribution becomes apparent. Although the majority of dihedral angles remain localized within the β-sheet basin, a greater dispersion toward adjacent conformational states is observed, reflecting enhanced thermal motion and increased backbone flexibility. Importantly, however, the β-sheet signature persists as the dominant structural motif, demonstrating that thermal agitation does not disrupt the primary supramolecular ordering. This resilience correlates with the energetic results reported earlier, which showed that peptide–peptide interactions remain strong despite a reduction in hydrogen-bond lifetimes at elevated temperatures.

The multilayer systems at 300 K, shown for the two- and three-layer configurations, display even more pronounced clustering within the β-sheet region compared to the monolayer case. The highly localized angular populations reflect restricted conformational freedom and enhanced stabilization of the extended peptide backbone when stacked into multilayer assemblies. This increased rigidity is consistent with the cooperative reinforcement of intermolecular contacts previously quantified through Coulomb and van der Waals contributions, and it confirms that vertical packing promotes a more constrained and ordered conformational landscape.

Taken together, the Ramachandran plots provide clear structural evidence that EF_4_K membranes retain β-sheet-like conformations across different thermal and organizational regimes. The persistence of this signature at elevated temperature highlights the thermal robustness of the monolayer assembly, while the sharpening of conformational populations in multilayer systems underscores the stabilizing effect of supramolecular stacking. These observations collectively support the conclusion that EF_4_K peptides form mechanically and thermodynamically resilient lamellar architectures, with conformational order maintained through a combination of strong intermolecular interactions and restricted backbone flexibility within the membrane environment.

## 4 Conclusions

In this work, we investigated the structural, energetic, and dynamical behavior of EF_4_K peptide membranes under varying temperature conditions and multilayer architectures using fully atomistic molecular dynamics simulations. The results demonstrate that EF_4_K peptides exhibit a strong intrinsic tendency to self-assemble into ordered β-sheet lamellar structures, maintaining robust supramolecular cohesion across a broad thermal range. Even at elevated temperatures, the monolayer system preserved its characteristic β-sheet signature, although with increased dynamic fluctuations and transient hydrogen-bond exchange, indicating a balance between structural integrity and thermal adaptability. A central finding of this study is the enhanced stability observed in multilayer configurations. The stacking of peptide sheets reinforced peptide–peptide interactions and reduced solvent exposure, leading to stronger electrostatic and van der Waals stabilization within the peptide framework. Confined water within multilayer systems exhibited longer hydrogen-bond lifetimes despite reduced hydration levels, suggesting selective retention of water molecules in structured interlamellar environments. These phenomena highlight the cooperative nature of intermolecular forces in promoting supramolecular rigidity and improving resistance to thermal perturbation. Overall, the EF_4_K nanomembranes presented a highly resilient structural profile, capable of maintaining order and mechanical cohesion under thermal stress, with multilayer systems providing superior stabilization through compact packing and solvent exclusion. These findings advance the understanding of bola-amphiphilic peptide assembly and highlight the potential of EF_4_K-based membranes as thermally robust biomaterials. Their capacity to form stable, layered architectures with confined and structured water suggests promising applications in nanobiotechnology, biofiltration, and bioelectronic interfaces where mechanical integrity, controlled hydration, and thermal resistance are essential.

## Supplementary Material



## Notes

Additional analyses that complement and validate the results; Equilibration behavior of the EF4K peptide membranes (1L, 2L, and 3L) at 300 K, including the convergence of total potential energy during the equilibration stage (Figure S1); statistical distribution of peptide–peptide hydrogen bonds for the production simulations of mono- and multilayer systems at 300 K (Figure S2); complete hydrogen-bond data set obtained using a relaxed geometric criterion (donor–H–acceptor angle cutoff of 40°), including average numbers of hydrogen bonds, lifetimes, and Gibbs free energies for peptide–peptide and peptide–solvent interactions across temperatures and stacking configurations (Table S1) (PDF)
