# Supplementary material for: Temperature Effects on the Structural Stability of EF4K Peptide Membranes: Insights into Mono- and Multilayer Architectures
Source: ACS Mater Au. 2026 Jan 2;6(2):437–49. doi: 10.1021/acsmaterialsau.5c00214 (PMC12983100; doi:10.1021/acsmaterialsau.5c00214)
Supplement: Supplementary file 1 [file mg5c00214_si_001.pdf]

SUPPORT INFORMATION MATERIAL

Temperature Effects on the Structural Stability of EF_4K Peptide Membranes: Insights into Mono- and Multilayer Architectures

Karinna Mendanha[1], Douglas Xavier de Andrade[2] and Guilherme Colherinhas[1]*

[1] Instituto de Física, Universidade Federal de Goiás, 74690-900, Goiânia, GO, Brazil.
2 Instituto Federal de Educação Ciência e Tecnologia de Goiás, 74968-755, Aparecida de Goiânia, GO, Brazil.
[*] corresponding author: gcolherinhas@ufg.br (GC)

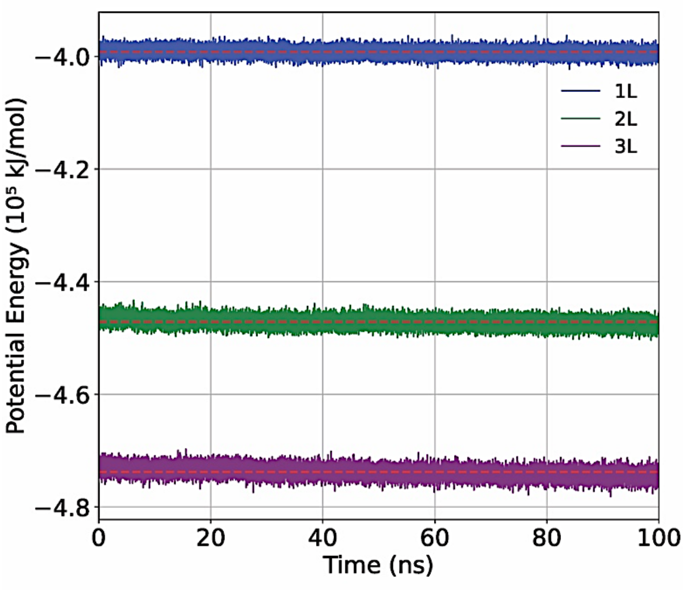

Figure S1: Convergence of structural and energetic observables during equilibration of 1L, 2L and 3L EF_4K peptide membranes at 300 K. Total potential energy profile converging to a stationary mean without systematic drift. (a) Model-1L, (b) Model-2L, and (c) Model-3L.

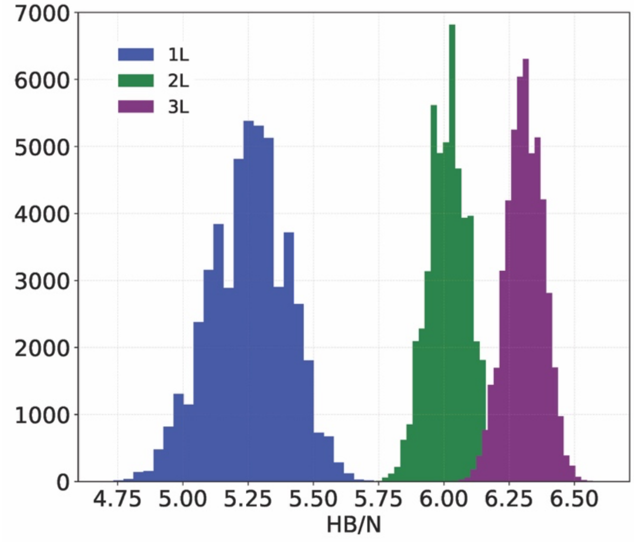

Figure S2: Histogram of peptide-peptide hydrogen-bonds for production MD simulation: (a) 1L, (b) 2L and (c) 3L EF_4K membranes at 300 K.

Table S1: Average number of hydrogen bonds, lifetimes (t), and Gibbs free energies (ΔG) for EF_4K systems at different temperatures and stacking configurations, reported per peptide ($r \leq 0.35$ nm and $\theta = 40^o$). Lifetimes (t) are given in nanoseconds (ns) and ΔG values in $kJ \cdot mol^{-1}$.

Model	***T***	***Pep-Pep***			***Pep-Sol***		
		HB	***t***	***ΔG***	***HB***	***t***	***ΔG***
1L	*250*	*5.2*	*1.82*	*23.1*	*15.9*	*0.18*	*17.5*
	270	*5.2*	*1.22*	*22.1*	*15.1*	*0.11*	*16.2*
	300	*5.6*	*0.80*	*21.1*	*13.5*	*0.05*	*14.3*
	320	*5.5*	*0.60*	*20.4*	*13.1*	*0.03*	*13.1*
	350	*5.8*	*0.40*	*19.4*	*11.8*	*0.02*	*11.8*
2L	*300*	*6.5*	*2.17*	*23.6*	*10.7*	*0.31*	*18.8*
3L	*300*	*6.9*	*2.98*	*24.5*	*9.4*	*0.58*	*20.3*
